# ﻿Three new species of the Neotropical genus *Smilidarnis* Andrade (Hemiptera, Membracidae)

**DOI:** 10.3897/zookeys.1174.103324

**Published:** 2023-08-08

**Authors:** Stuart H. McKamey

**Affiliations:** 1 Systematic Entomology Laboratory, Agricultural Research Service, U.S. Department of Agriculture, c/o National Museum of Natural History, P.O. Box 37012, Washington, D.C. 20013, USA c/o National Museum of Natural History Washington, D.C. United States of America

**Keywords:** Brazil, Ecuador, Neotropical, new species, Peru

## Abstract

*Smilidarnisduocornus***sp. nov.**, *S.erwini***sp. nov.**, and *S.robustus***sp. nov.** are described, illustrated, and included in a key to the five species here recognized in *Smilidarnis*. One member of this genus, from Ecuador (new country record for genus), is *S.erwini*, which differs from its congeners in having distinct coloration and being of intermediate size in terms of overall body length and the relative length of the lateral apical spines. *Smilidarnisrobustus* (from Peru) and *S.duocornus* (from Brazil) differ from the other species of *Smilidarnis* in having the pronotum bearing a pair of suprahumeral spines.

## ﻿Introduction

There are over 3,500 species of Membracidae worldwide (Bartlett et al. 2018), and very few of those cannot be placed to subfamily based on a combination of leg chaetotaxy, wing venation, and degree of forewing coverage by the pronotum. Of the six genera that McKamey (1998) listed as Membracidae*incertae sedis*, five have been subsequently referred elsewhere: *Antillotolania* Ramos and *Deiroderes* Ramos to Stegaspidinae (Cryan and Deitz 2002), *Euwalkeria* Goding and *Holdgatiella* Evans to Nicomiini (Albertson and Dietrich 2005), and *Megsaloschema* Buckton to Centrotinae (Wallace and Deitz 2004). Only the listed genus *Smilidarnis* Andrade remained unplaced. Since McKamey’s catalog (1998), however, three other unplaced genera have been described, all of which have a pronotum that does not project posteriorly over the scutellum: *Togotolania* Cryan & Deitz (2002), *Smergotomia*Dietrich (2008), and *Problematode*Gaiani (2017). The genus *Brachytalis* Metcalf & Bruner, which was previously placed in Nessorhinini (Deitz 1975), has since been referred to Membracidae*incertae sedis* (Wallace and Deitz 2004). Flórez-V. (2019) referred *Problematode* to Stegaspidinae based on nymph morphology, bringing the total number of unplaced membracid genera back up to four: *Togotolania*, *Smergotomia*, *Brachytalis*, and *Smilidarnis*.

**Figure 1.**
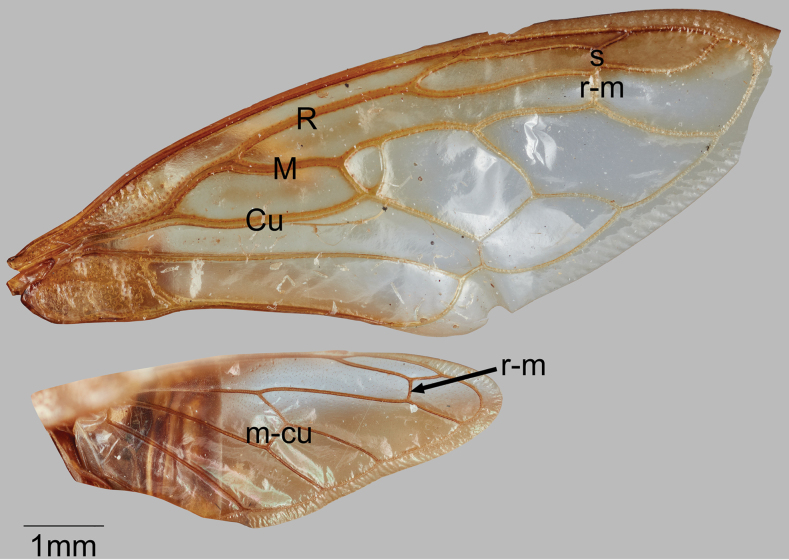
*Smilidarnisrobustus* sp. nov., forewing and hind wing.

Andrade (1989) described the genus *Smilidarnis* with two new species, *S.fasciatus* (from Peru) and *S.concolor* (from Brazil), leaving it as unplaced Membracidae because it has features of both Smiliinae and Darninae. Like many species of Smiliinae, the forewing veins R and M are basally fused (Fig. 1). Like Darninae, *Smilidarnis* has crossvein s (between the branches of the radial veins), two m-cu crossveins in the forewing, and one r-m in the hind wing (Fig. 1). However, resolving the relationship of *Smilidarnis* to other treehoppers is further complicated by the results of Dietrich et al. (2017), who found that in some analyses both Darninae and Smiliinae are polyphyletic. In the most recent phylogenetic study of Membracidae (Evangelista et al. 2017), which used seven gene sequences, *Smilidarnis* was the sister group of Ceresini (Smiliinae). An unlabeled specimen of *S.fasciatus* is held by The Natural History Museum in London, England (pers. observ.), and a color photograph of the holotype is available online at http://treehoppers.insectmuseum.org/public/figure/show_zoom/69718 (Deitz and Wallace 2010 [and updates]).

## ﻿Materials and methods

In quoting labels, quotation marks separate labels and a vertical line separates lines on a label.

Terminology for general morphology, forewing venation (except crossvein s), and leg chaetotaxy follows Deitz (1975).

A Leica MZ12 stereomicroscope was used to examine structures. The body length was measured using a digital micrometer, as was the relative distances of the eye (inner margin) and ocelli (centers). A manual 5 mm micrometer was used and to determine ratios between other, shorter distances.

The abdomen was detached, macerated in a 10% KOH solution at room temperature for 24 h, bathed in water, then acetic acid to stop the reaction. After dissection, structures were stored in a glass microvial containing glycerin and pinned beneath the specimen.

Images were taken with a Canon 5Dsr camera with an adjustable 65mm lens. Photos were taken using Capture One Pro version 10.1.2, 64 bit, build 10.1.2.23 imaging software, aided by CamLift version 2.9.7.1. The specimen was lit using two adjustable Dynalite MH2050 RoadMax flash heads, each attached to a Manfrotto 244 arm. The light was diffused using a lampshade-style cone of translucent paper between the specimen and light sources. After individual focal planes were photographed, they were compiled into a single, composite image using Zerene Stacker - USDA SI-SEL Lab Bk imaging system, version 1.04, build T201706041920. Stacked images were enhanced and edited in Adobe Photoshop CSS Extended version 12.0. The scale bars were generated through Photoshop directly from the metadata of the photo.

The holotype of *S.robustus* is deposited in the U.S. National Museum of Natural History, Smithsonian Institution, Washington, DC (USNM). The holotype of *S.erwini* is deposited in the Museo de la Escuela Politécnica Nacional, Quito, Ecuador (EPNC). The holotype of *S.duocornus* is deposited in the Instituto Nacional de Pesquisas da Amazonia, Coleção Sistemática da Entomologia, Manaus, Brazil (INPA).

## ﻿Results

### ﻿ Smilidarnis

Taxon classificationAnimaliaHemipteraMembracidae

Andrade 1989: 695

C64FD89C-B8B3-5D11-8624-96E983168D56

#### Type species.

*S.fasciatus*Andrade 1989: 696.

#### Diagnosis.

Pronotum with 3 posterior spines; forewing (Fig. 1) with veins R, M, and Cu fused basally, R and M strongly divergent at about 1/3 distance of wing length, with crossvein s (between radial veins), 1 r-m and 2 m-cu crossveins; hind wing (Fig. 1) with 1 r-m and 1 m-cu crossvein; metathoracic trochanters without apposed processes; metathoracic tibia chaetotaxy variable.

#### Distribution.

South America.

#### Notes.

The only discrepancy between the generic description by Andrade (1989) and the newly described species is that he reported metathoracic tibial cucullate rows II and III double and, by implication, row I single. This pattern is also found in Ceresini (Deitz 1975). In contrast, *S.duocornus* has row I absent and *S.robustus* has rows I and III double and row II absent. Among Darninae, some Darnini and some Hemikypthini lack tibial cucullate setae row I, but only some Hemikypthini lack row II (Deitz 1975). Consequently, leg chaetotaxy does not provide evidence resolving the relationship of *Smilidarnis* to other treehoppers.

The forewing venation of the three new species described here matches that of *S.fasciatus*, in that the veins R_4+5_ and M_1+2_ are distally separate; in *S.concolor* Andrade, those veins are confluent for short distance before the apex. Among all five species of *Smilidarnis*, only *S.concolor* has the forewing veins R and M fused then separated preapically (as in Smiliinae). *Smilidarnisduocornus* and S. *robustus* resemble each other in the shape of the frontoclypeus and pronotum, as do the other three species resemble each other in these respects.

In some membracids, the presence of abdominal fossae or digitate processes in adults (e.g., see Deitz 1975: fig. 3a) are indicators of scoli in nymphs; the absence of these suggests that the nymphs of *Smilidarnis* probably lack scoli. Further collecting of these exceedingly rare treehoppers and happenstance rearing is needed to elucidate their biology and immatures.

### ﻿Key to species

**Table:** 

1	Pronotum with pair of stout suprahumeral spines	**2**
–	Pronotum without suprahumeral spines	**3**
2	Breadth across suprahumeral spines distinctly greater than breadth across posterior lateral spines (Fig. 2)	***S.duocornus* sp. nov.**
–	Breadth across suprahumeral spines subequal, slightly less than breadth across posterior lateral spines (Fig. 14)	***S.robustus* sp. nov.**
3	Pronotum posteriorly with tips but not bases of lateral spines black; forewing with veins R and M distally fused then separated preapically	***S.concolor* Andrade**
–	Pronotum posteriorly with bases of lateral spines black; forewing with veins R and M not fused preapically	**4**
4	Pronotum with central apical spine pale throughout; head vertex with ventrolateral margins and frontoclypeus forming evenly convex curve (Fig. 9)	***S.erwini* sp. nov.**
–	Pronotum with central apical spine black in distal third; head vertex with ventrolateral margins straight and frontoclypeus forming an angle	***S.fasciatus* Andrade**

### ﻿ Smilidarnis duocornus sp. nov.

Taxon classificationAnimaliaHemipteraMembracidae

0292BFB4-28F2-5546-BEA9-B09FF7E0DEB7

https://zoobank.org/83FF1199-024D-4397-A31F-A942B5B39E81

Figs 2–7

#### Diagnosis.

Pronotum with pair of suprahumeral spines; pronotum broader across suprahumeral spines than across posterior spines.

#### Description of female.

**Figures 2–7.**
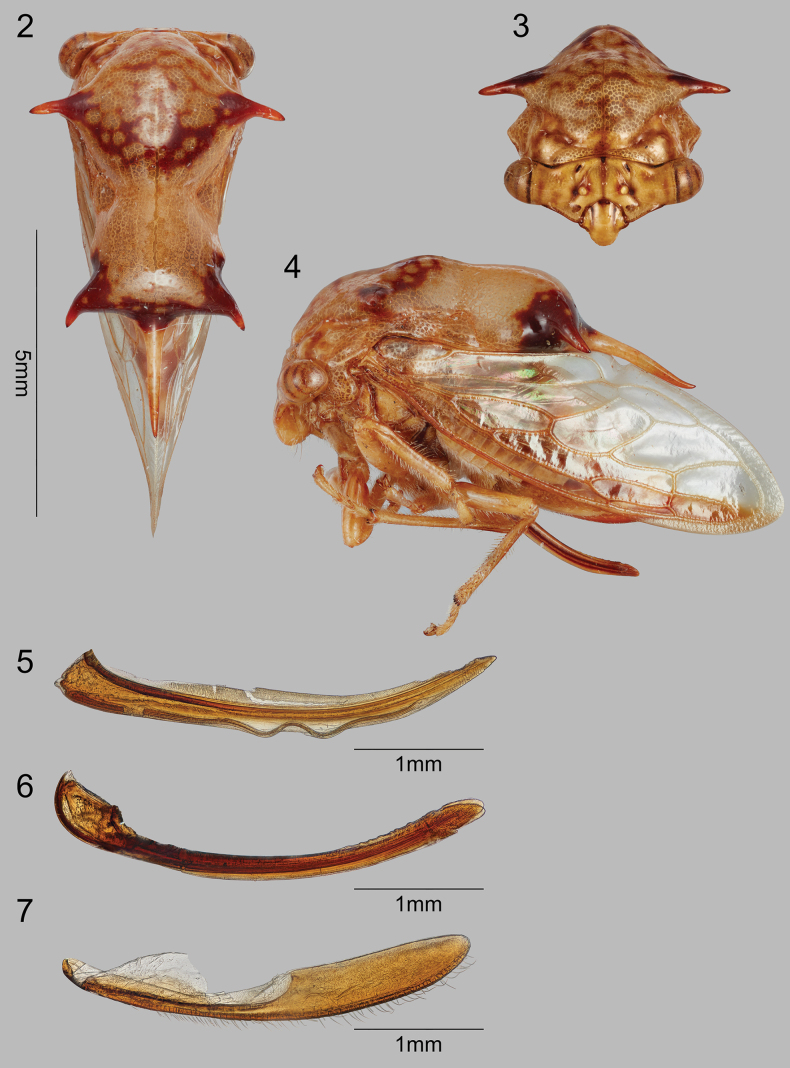
*Smilidarnisduocornus* sp. nov. **2–4** habitus in dorsal, anterior, and lateral views, respectively **5–7** valvifers 1, 2, and 3, respectively.

Dimensions (mm). Length of pronotum 7.5; length including wings in repose 9.5; width across suprahumeral spine apices 4.5; width across posterolateral spine apices 3.0; height in anterior view 4.3. Head (Fig. 3). Ocelli circular, below imaginary midline between eyes; distance to eye 1.2× distance between ocelli; vertex with depression ventrolaterally adjacent to ocelli, pair of narrow diagonal depressions dorsally, with distinct sutures and frontoclypeus extending ventrally beyond vertex ventrolateral margins. Pronotum (Figs 2–4) with pair of stout suprahumeral spines; pronotum distinctly broader across suprahumeral spines than across posterior spines; weakly elevated immediately behind suprahumeral spines, this portion roughly trapezoidal in lateral view, abruptly narrowed laterally and expanding again to apical portion that bears pair of widely separated, stout lateral spines directed posteriorly and slender middle spine. Forewing (as in Fig. 1) R and M not confluent for short distance near apex. Leg chaetotaxy. Femora lacking cucullate setae and spines; metathoracic tibia row I lacking cucullate setae, row II cucullate setae sparse in single line, row III cucullate setae dense, single row basally, double row distally. Abdominal terga without pits, fossae, or digitate processes. Color. Head pale brown except darker in depressions and along sutures of frontoclypeus; pronotum pale brown throughout except dark brown mottling in anterior half, at bases of suprahumeral spines, and posteriorly across lateral spines.

#### Female terminalia.

Sternite VII broadly emarginate medially (as in Fig. 17); valvula I (Fig. 5) long, apex subacute; valvula II (Fig. 6) in lateral view with dorsal margin linear in basal 2/3, then minutely sinuate, apex rounded; valvula III (Fig. 7) long, apex rounded, bearing macrosetae along entire ventral margin.

#### Male.

Unknown.

#### Material examined.

***Holotype*** ♀ (INPA) with labels “BRAZIL: Rondonia. 62 | km SW Ariquemes, nr | Fzda. Rancho Grande | 5–17-X-1993 JE Eger | MV & Black Lights” and a red “HOLOTYPE | Smilidarnis | duocornus | S.H. McKamey”.

#### Etymology.

The species is a Latin adjective derived from “duo” for two, and “cornu” for horn, referring to the pair of stout suprahumeral spines.

#### Notes.

The pronota of *S.duocornus* and *S.robustus*, which are both only known from a female holotype, closely resemble each other and have similar coloration. In some treehoppers, such as *Quinquespinosaseptamacula*McKamey (2023), color and surprahumeral spine length is variable, so these differences alone might not suffice for species recognition. Nevertheless, these are considered separate species for several reasons: *S.sobustus* is almost 3 mm larger; its posterior portion is more elevated, and the apical lateral spines are directed more laterally. Futhermore, the setation of valvifer III is distinct; bearing macrosetae along ventral margin in S. *duocornus* (Fig. 7), in contrast to that of *S.robustus*, which bears fine hairlike setae on entire ventral half (Fig. 20).

### ﻿ Smilidarnis erwini sp. nov.

Taxon classificationAnimaliaHemipteraMembracidae

B5E38D40-232C-5BBC-8AC1-1C03E9D715F7

https://zoobank.org/DF05FB0E-5FC5-47EE-9F82-3160A941B6A4

Figs 8–13

#### Diagnosis.

Pronotum without pair of suprahumeral spines; vertex of head with four dark lines, outer pair adjacent to eyes and inner pair ventrally converging.

#### Description of male.

**Figures 8–13.**
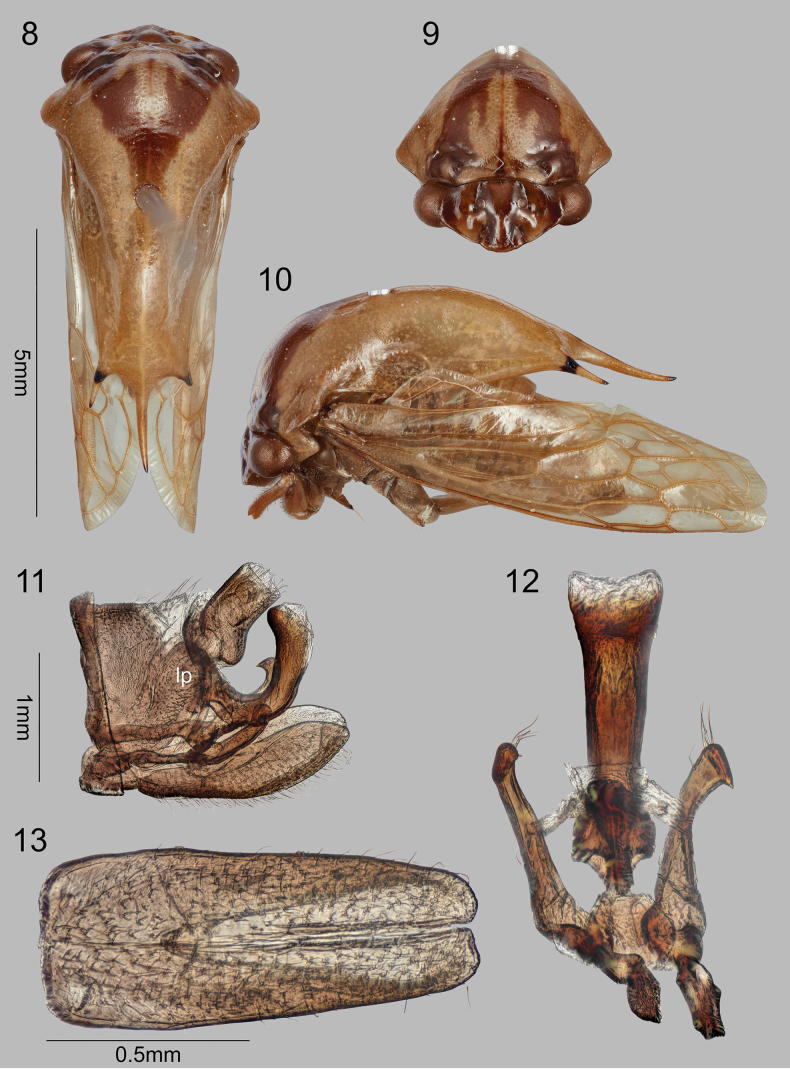
*Smilidarniserwini* sp. nov. **8–10** habitus in dorsal, anterior, and lateral views, respectively **11** pygofer, styles, and aedeagus, lateral view **12** aedeagus and styles, anterior view **13** subgenital plate, ventral view. *lp* lateral plate.

Dimensions (mm). Length of pronotum 8.5; length including wings in repose 10.0; width across humeri 3.7; width across posterolateral spine apices 1.5; height in anterior view 4.2. Head (Fig. 9). Ocelli circular, above imaginary middle line between eyes; distance to eye 0.8× distance between ocelli; vertex with adjacent depression ventrolaterally; frontoclypeus with indistinct sutures and evenly convex in line with ventrolateral margins. Pronotum (Figs 8–10). Humeral angles rounded, only slightly produced laterally from wing bases. Forewing (as in Fig. 1) R and M not confluent for short distance near apex. Leg chaetotaxy. Femora of pro- and metathorax lacking cucullate setae and spines (other femora missing); metathoracic tibiae missing. Abdominal terga without pits, fossae, or digitate processes.

#### Male terminalia

(Figs 11–13). Pygofer (Fig. 11) including lateral plate subquadrate, lateral plate unarmed, ovate, pilose throughout, subgenital plate (Fig. 13) fused basally, its sides subparallel, slightly narrowing distally, length in ventral view about 2.3× width; style (Figs 11, 12) recurved with short acute apex; aedeagus (Figs 11, 12) U-shaped in lateral view, shaft subparallel in anterior and lateral views, lacking dentae or other texture on distoanterior portion; gonopore dorsal. Color. Pale brown throughout except 4 dark lines on head vertex (outer pair adjacent to eyes, inner pair overlapping ocelli and ventrally convergent), 1 large median, dark brown, anteriorly divergent, V-shaped mark on pronotal metopidium, all 3 apical spines with bases and apices black but pale at mid length.

**Female.** Unknown.

#### Material examined.

***Holotype*** ♂ (EPNC) with labels “ECUADOR: ORELLANA: Reserva Etnica Waorani, 1 | km S. OnkoneGare Camp Transect Ent 216.3m | 15-Jan-94 00°39'25.7"S, 076°27'10.8"W T.L | Erwin: et al Fogging Terre firme forest Lot 579”, and a red “HOLOTYPE | Smilidarnis | erwini | S.H. McKamey”. All legs are missing or badly damaged but the specimen, excluding its abdominal dissection, is otherwise intact.

#### Etymology.

The species is named to honor Terry Erwin for his innovative and revolutionary method of collecting insects through insecticidal fogging of the forest canopy, which has collected many new species, including this one.

#### Notes.

The pronotal apex of *S.erwini* resembles that *of S.fasciatus* in having the bases and tips of the posterolateral spines black. In *S.fasciatus*, however, in addition to other differences, those lateral spines are nearly as long as the middle spine, in comparison to *S.erwini*, in which they are about half the length of the middle spine, and *S.concolor*, in which they are even more reduced.

### ﻿ Smilidarnis robustus sp. nov.

Taxon classificationAnimaliaHemipteraMembracidae

71FDE069-5352-5E13-A263-0138ACB9D7B0

https://zoobank.org/DDA76B99-A2D7-4802-9F6A-798079CE5D0C

Figs 1 , 14–20

#### Diagnosis.

Pronotum with pair of suprahumeral spines; pronotal width across posterior spines slightly greater than width across suprahumeral spines.

#### Description of female.

**Figures 14–20.**
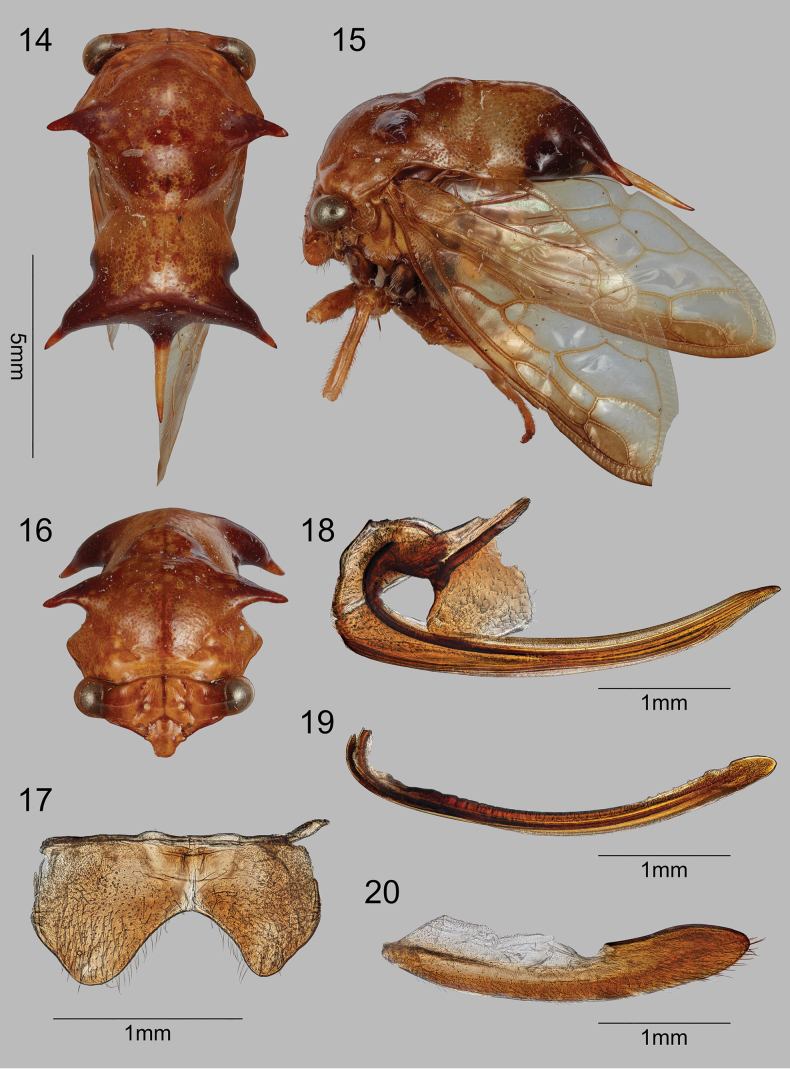
*Smilidarnisrobustus* sp. nov. **14–16** habitus in dorsal, lateral, and anterior views, respectively **17** abdominal segment VII, ventral view **18–20** valvifers 1, 2, and 3, respectively.

Dimensions (mm). Length of pronotum 10.3; length including wings in repose 12.4; width across suprahumeral spine apices 5.0; width across posterolateral spine apices 5.8; height in anterior view 5.1. Head (Fig. 16). Vertex wider than tall, glabrous, without irregular ridges, dorsal margin weakly convex, lateral margins straight; ocelli circular, below imaginary middle line between eyes; distance to eye 1.3× distance between ocelli; midline and frontoclypeal sutures prominent; frontoclypeus extending ventrally beyond vertex ventrolateral margins, with distinct sutures. Pronotum (Figs 14–16). Suprahumeral spines present with robust bases, projecting laterally and slightly ventrally; weakly elevated immediately behind suprahumeral spines, this posterior portion roughly trapezoidal in lateral view, abruptly narrowed laterally and expanding again to apical portion that bears pair of widely separated stout lateral spines and middle slender spine; lateral pair with apices broader than span of suprahumeral spines and directed posterolaterally, middle spine directed posteriorly and not attaining forewing vein M_3+4_. Forewing (Fig. 1) R and M not confluent for short distance near apex. Leg chaetotaxy. Femora lacking cucullate setae and spines; metathoracic tibia row I cucullate setae double, row II absent, row III cucullate setae double.

**Female terminalia.** Sternite VII broadly, smoothly emarginate medially (Fig. 17); valvula I long, apex subacute (Fig. 18); valvula II (Fig. 19) in lateral view with dorsal margin linear in basal 2/3, then bearing 4 minute dentae followed by weak irregular expansion, apex rounded; valvula III (Fig. 20) long, apex rounded broadly, bearing fine hairlike setae on entire ventral half. Abdominal terga without pits, fossae, or digitate processes. Color. Pronotum burnt orange throughout except dark brown on suprahumeral spines, apex including lateral spines, this apical darkness interrupted by pair of subcircular pale spots on either side of the middle spine (visible in posterior view only); forewing anteriorly with amber tint, otherwise hyaline; ventrally concolorous with pronotum except black meso- and metathoracic coxae and pleural area between them.

**Male.** Unknown.

#### Material examined.

***Holotype*** ♀ (USNM) with labels “Shapajilla, | Peru | May, 1939”, “WDFunkhouser | Collection | 1962”, “Omalon [sic] | sp. nov.”, “Deitz Research | 71-350a ♀”, “CHD Research | #98-0004”, and a red “HOLOTYPE | Smilidarnis | robustus | S.H. McKamey”. There are several parts missing from the holotype: prothoracic left tibia and tarsi (the right is covered in glue), mesothoracic left tarsi (the right is covered in glue), and the metathoracic left trochanter to tarsi. The holotype has the left wing with an aberration of a thin third m-cu crossvein.

#### Etymology.

The species epithet is a masculine adjective referring to the overall robustness of this species’ pronotum.

## Supplementary Material

XML Treatment for Smilidarnis

XML Treatment for Smilidarnis duocornus

XML Treatment for Smilidarnis erwini

XML Treatment for Smilidarnis robustus

## ﻿Additional information

### Author ORCIDs

Stuart H. McKamey https://orcid.org/0000-0002-9617-0594

### Data availability

All of the data that support the findings of this study are available in the main text or Supplementary Information.
